# Biochemical Phenotypes to Discriminate Microbial Subpopulations and Improve Outbreak Detection

**DOI:** 10.1371/journal.pone.0084313

**Published:** 2013-12-31

**Authors:** Alicia Galar, Martin Kulldorff, Wallis Rudnick, Thomas F. O'Brien, John Stelling

**Affiliations:** 1 WHO Collaborating Centre for Surveillance of Antimicrobial Resistance, Department of Medicine, Brigham and Women's Hospital, Harvard Medical School, Boston, Massachusetts, United States of America; 2 Department of Population Medicine, Harvard Medical School and Harvard Pilgrim Health Care Institute, Boston, Massachusetts, United States of America; 3 Department of Medicine, Mount Sinai Hospital, Toronto, Ontario, Canada; The University of Tokyo, Japan

## Abstract

**Background:**

Clinical microbiology laboratories worldwide constitute an invaluable resource for monitoring emerging threats and the spread of antimicrobial resistance. We studied the growing number of biochemical tests routinely performed on clinical isolates to explore their value as epidemiological markers.

**Methodology/Principal Findings:**

Microbiology laboratory results from January 2009 through December 2011 from a 793-bed hospital stored in WHONET were examined. Variables included patient location, collection date, organism, and 47 biochemical and 17 antimicrobial susceptibility test results reported by Vitek 2. To identify biochemical tests that were particularly valuable (stable with repeat testing, but good variability across the species) or problematic (inconsistent results with repeat testing), three types of variance analyses were performed on isolates of *K. pneumonia*: descriptive analysis of discordant biochemical results in same-day isolates, an average within-patient variance index, and generalized linear mixed model variance component analysis. Results: 4,200 isolates of *K. pneumoniae* were identified from 2,485 patients, 32% of whom had multiple isolates. The first two variance analyses highlighted SUCT, TyrA, GlyA, and GGT as “nuisance” biochemicals for which discordant within-patient test results impacted a high proportion of patient results, while dTAG had relatively good within-patient stability with good heterogeneity across the species. Variance component analyses confirmed the relative stability of dTAG, and identified additional biochemicals such as PHOS with a large between patient to within patient variance ratio. A reduced subset of biochemicals improved the robustness of strain definition for carbapenem-resistant *K. pneumoniae*. Surveillance analyses suggest that the reduced biochemical profile could improve the timeliness and specificity of outbreak detection algorithms.

**Conclusions:**

The statistical approaches explored can improve the robust recognition of microbial subpopulations with routinely available biochemical test results, of value in the timely detection of outbreak clones and evolutionarily important genetic events.

## Introduction

Clinical and public health microbiology laboratories worldwide constitute an invaluable resource for monitoring spread of antimicrobial resistance and other emerging microbial threats [1], [2]. Yet findings from these laboratories are underutilized for the systematic monitoring of evolving microbial populations including the early recognition, crucial for effective containment interventions, of new mutation events, emergent strains, and outbreaks [3], [4].

Although surveillance is a major part of infection control programs, the processes of surveillance activities in most hospitals are largely unstudied [5], and available microbiology results ineffectively utilized. In public health agencies and healthcare facilities, outbreak detection depends primarily on incidental observations of unexpected morbidity/mortality or monitoring increased frequencies of a few organisms or organism subtypes of interest (*e.g.* methicillin-resistant *Staphylococcus aureus*, carbapenem-resistant *Enterobacteriaceae*). This requires the sustained efforts of infection control staff with limited time and data management tools, misses events meriting investigation, and exhibits delays in detection when compared to a statistical approach [1], [6]. Previous work suggests that the use of routinely available antimicrobial resistance phenotypes can improve the sensitivity and specificity of outbreak detection algorithms through more refined definition of microbial subpopulations [1], [6], [7].

In this work, we explore the utility of biochemical phenotypes [8] to the same end – improved recognition of microbial clones on the basis of biochemical tests such as glucose and urease used by clinical laboratories for the routine identification of microorganisms. While most biochemical tests are relatively consistent across all isolates of a given bacterial species (either >95% positive or >95% negative) and thus useful for species identification, others exhibit greater variability across the species [9] and thus are potentially valuable for strain identification below the species level. A well-characterized example is the finding that strains of *Escherichia coli* O157:H7, often associated with foodborne disease outbreaks and hemolytic uremic syndrome, typically do not ferment sorbitol (“sorbitol-negative *E. coli*”) in contrast to most *E. coli* [10], [11]. As a result of this observation, sorbitol fermentation has become a practical test useful for the routine screening of these enterohemorrhagic strains. Some specialized commercial phenotyping systems, such as PhPlate AB’s PhenePlate system or Biolog’s Phenotype MicroArray exploit this variation within a species for epidemiological and research purposes, in contrast to the much more widespread bioMérieux’s Vitek product or Siemens MicroScan instrument which focus on microbial identification at the species level.

Variability in biochemical results within a species can be due to one of two factors: 1. true reproducible differences between distinct strains; and 2. inconsistent test findings due to variability in test performance factors (inoculum, test conditions, *etc.*) or in biological expression of the phenotype. In this study we apply statistical approaches for the analysis of biochemical phenotypes of *Klebsiella pneumoniae* for the recognition of specific biochemicals of greatest and least value in defining phenotypically distinct strains, and explore as a practical application of the conclusions how this information could be utilized to study the evolution of carbapenem resistance in a tertiary care hospital and to improve outbreak detection.

## Methods

### Ethics Statement

This study was approved by the Brigham and Women's Hospital (BWH) Human Research Committee and no informed consent was required.

### Study Population and Datasets

Microbial identification and antimicrobial susceptibility test results for *K. pneumoniae* from January 2009 through December 2011 of a 793-bed academic center were imported from the Vitek® 2 system (bioMérieux, Marcy l’Etoile, France) into WHONET [12]–[15], a free software used in over 110 countries developed by our group, the WHO Collaborating Centre for Surveillance of Antimicrobial Resistance. Variables evaluated included patient identifier, location, collection date, organism, and biochemical and antimicrobial susceptibility test results.

The Vitek 2 ID-GNB Panel uses a set of 47 biochemical tests plus a negative control for the identification of Gram-negative bacteria (Table 1). Results are recorded as 0 for negative results and 1 for positive results. These 48 values are compacted by the Vitek by combining triplets of positive/negative binary results into a 16-digit Vitek “bionumber”. In order to analyze the individual biochemical results, this compaction process was reversed in Microsoft Excel to extract the bionumber into the 48 individual component results. Statistical analyses were performed using WHONET 5.6, SaTScan 9.0, SAS version 9.2 and SPSS 15.0.

**Table 1:** Frequency of positive test results for patient first isolates of *Klebsiella pneumoniae* considering the 47 biochemical tests and negative control included in the Vitek 2 ID-GNB Panel.

Biochemical test	Biochemical code	Positive percentage(95% CI^1^)	Weighted AverageWithin-Patient Variance	GLIMMIX CPE^2^
Negative control	N_CON	0 (0.0000–0.0015)	0.0000	n/c
Lipase	LIP	0 (0.0000–0.0015)	0.0000	n/c
L-Arabitol	lARL	0 (0–0.2)	0.0000	n/c
D-Glucose	dGLU	100 (99.8–100)	0.0000	n/c
Glu-Gly-Arg-Arylamidase	GGAA	0 (0.0000–0.0015)	0.0001	0.2208
Ala-Phe-Pro-Arylamidase	APPA	0 (0.0000–0.0015)	0.0001	n/c
Glutamyl Arylamidase pNa	AGLTp	0 (0–0.2)	0.0001	n/c
Beta-Alanine Arylamidase pNa	BAlap	0.2 (0.1–0.5)	0.0002	0.0000
D-Manitol	dMAN	99.7 (99.4–99.9)	0.0002	n/c
Beta-N-Acetyl-Galactosaminidase	NAGA	0.2 (0.1–0.5)	0.0003	n/c
Alpha-Galactosidase	AGAL	99.9 (99.6–100)	0.0003	0.4315
Beta-Glucosidase	BGLU	99.6 (99.2–99.8)	0.0003	n/c
D-Mannose	dMNE	99.9 (99.6–1)	0.0004	0.1536
Phosphatase	PHOS	99.6 (99.3–99.8)	0.0004	3.9521
D-Cellobiose	dCEL	99.8 (99.5–99.9)	0.0005	n/c
D-Trehalose	dTRE	99.8 (99.6–100)	0.0006	n/c
Saccharose/Sucrose	SAC	99.5 (99.1–99.7)	0.0007	n/c
Alpha-Glucosidase	AGLU	0.9 (0.5–1.3)	0.0007	n/c
H_2_S Production	H2S	0.4 (0.2–0.7)	0.0009	n/c
Beta-Galactosidase	BGAL	99.2 (98.8–99.5)	0.0012	n/c
ELLMAN	ELLM	1.2 (0.8–1.7)	0.0012	n/c
Fermentation/Glucose	OFF	99.5 (99.1–99.7)	0.0013	n/c
D-Sorbitol	dSOR	97.4 (96.7–98.0)	0.0014	n/c
Citrate-sodium	CIT	98 (97.4–98.5)	0.0015	3.4833
Palatinose	PLE	98.9 (98.4–99.2)	0.0015	3.4942
Ornithine Decarboxylase	ODC	1.6 (1.1–2.2)	0.0017	n/c
D-Maltose	dMAL	98.1 (97.4–98.6)	0.0020	3.4748
Malonate	MNT	93.7 (92.6–94.6)	0.0022	4.4685
Beta-Glucuronidase	BGUR	1.8 (1.3–2.4)	0.0022	n/c
Beta-N-Acetyl-Glucosaminidase	BNAG	2 (1.5–2.6)	0.0023	n/c
Beta-Xylosidase	BXYL	95.5 (94.6–96.2)	0.0026	n/c
Urease	URE	94.7 (93.7–95.5)	0.0032	3.8876
Lysine Decarboxylase	LDC	94.7 (93.8–95.6)	0.0034	3.7809
5-Keto-D-Gluconate	5KG	12.8 (11.5–14.2)	0.0039	4.4121
Adonitol	ADO	86.5 (85.1–87.8	0.0050	4.4025
L-Lactate Assimilation	ILATa	6.3 (5.4–7.3)	0.0052	3.1058
L-Pyrrolydonyl-Arylamidase	PyrA	96 (95.1–96.7)	0.0054	1.9750
Coumarate	CMT	11.8 (10.5–13.1)	0.0063	3.5695
L-Histidine Assimilation	lHISa	9.1 (8.0–10.3)	0.0066	3.1588
L-Lactate Alkalinisation	lLATk	95.4 (94.5–96.2)	0.0067	1.8028
2,4-Diamino-6,7-DiisopropylpteridineResistance	O129R	87.9 (86.5–89.1)	0.0075	2.7097
L-Malate Assimilation	IMLTa	11.6 (10.4–12.9)	0.0076	3.3123
D-Tagatose	dTAG	36.5 (34.6–38.4)	0.0082	4.7684
L-Proline Arylamidase	ProA	17.3 (15.8–18.8)	0.0084	3.5177
Gamma-Glutamyl-Tranferase	GGT	87.6 (86.2–88.8)	0.0122	2.5210
Glyicine Arylamidase	GlyA	31.8 (29.9–33.6)	0.0195	2.5966
Tyrosine Arylamidase	TyrA	52.3 (50.3–54.2)	0.0217	2.7779
Succinate Alkalisation	SUCT	43.5 (41.5–45.4)	0.0219	2.6267

^1^ Clopper-Pearson 95% Confidence Interval calculated with SAS.

^2^ Covariance Parameter Estimates (CPE), n/c = not convergence.

### Variance Analyses

To identify biochemical tests that were particularly valuable (stable with repeat testing, but good variability across the species) or problematic (inconsistent results with repeat testing), three types of variance analyses were performed: 1. Same-day isolates discordance analysis; 2. Average within-patient variance index; and 3. Generalized linear mixed model variance component analysis. Patients with only a single isolate of *K. pneumoniae* during the study period were excluded from these variance analyses.

#### Same-day isolates discordance analysis

Since the focus of this work is the characterization of variability with repeat testing (and not strain acquisition or mutation over time), a relevant exploratory analysis is the comparison of biochemical results of patient isolates which were collected on the same day. In this analysis, a data subset was created with two isolates from any patient with two or more *K. pneumoniae* isolates on the first day that this organism was found. For each biochemical, it was ascertained for each patient whether the two results were concordant (both positive or both negative) or discordant (one positive and one negative). For each biochemical, the proportion of patients with discordant results was tabulated.

#### Average within-patient variance index

To more fully utilize results from all isolates, an average within-patient variance index was calculated for each biochemical in the following way. Within-patient variance for each biochemical was calculated separately for each patient with 2 or more isolates using the binomial variance formula p(1−p)/n, were p = proportion of positive results for this patient, and n = number of isolates for this patient. For example, if a patient has 4 isolates of *K. pneumoniae* and three of these are dTAG-positive and one negative, then the dTAG within-patient variance for this patient would be 0.75 * 0.25/4 = 0.046875. These patient-specific variances were then averaged across all patients and weighted by the number of isolates for each patient to generate a weighted average within-patient variance for each biochemical across the patient population.

#### Between patient variance component

To be useful as an epidemiological marker, it is not only important to have concordance within patients, but there must also be variability between patients. In statistical terms, we want the within patient variance component to be small and the between patient variance component to be large. For continuous variables, simple and multiple linear regression can be used to estimate the variance of test results, but for the dichotomous biochemical test results studied here, generalized linear mixed models are more appropriate. As implemented within the SAS GLIMMIX procedure for binomial data, we calculated the covariance parameter estimate (CPE) for the between patient variance component. A large CPE indicates more variance between patients with is an important indicator if the biomedical test is to be useful as an epidemiological marker. If biochemical tests results are as similar between patients as within patients, the CPE is zero, indicating that it does not provide any information to differentiate between patients. This would happen if, for example, a test is positive with probability p independent of all other tests and regardless of the patient it comes from. Calculations were done using SAS PROC GLIMMIX [16], [17]. For biomedical tests with small within and small between patient variance, GLIMMIX did not converge, but these are situations when the biomedical tests provides little discriminatory information as it is almost always positive or almost always negative. Note that, when we consider between patient variance it is between patients that all have *K. pneumoniae.* Our results are not relevant to the ability to differentiate between patients with different pathogens.

### Cluster Detection

The value of a reduced subset of biochemicals for improving outbreak detection was evaluated with the use of WHONET-SaTScan. SaTScan™ [18] is a free software used for the detection of statistical clusters in space, in time, or in space and time. Previous work has demonstrated the value of the SaTScan prospective space-time permutation scan statistic for detecting clusters in routine laboratory data using collection date as the time variable and one of the following as the “spatial” variable: latitude and longitude [19], medical ward and service [6], resistance phenotype [1], and serotype [19]. For this analysis, patients’ first isolates of *K. pneumoniae* in a 365-day window were studied in a simulated prospective analysis run from 1 January 2010 through 31 December 2011 using biochemical phenotype as the “spatial” variable, collection date as the temporal variable, maximum cluster length of 60 days, 365 days of baseline data, and 9,999 Monte Carlo simulations. Signals with a recurrence interval (roughly the inverse of the p-value) of 365 days or greater were considered significant for alert purposes, which means that under the null hypothesis of no outbreaks the expected number of signals would be one during a one year period.

## Results

4,200 isolates of *K. pneumoniae* were identified from 1 January 2009 to 31 December 2011 from 2,485 patients. While the majority of patients (67.5%) had only a single isolate of *K. pneumoniae* during the study period, 32.5% of patients had two or more isolates, and this portion was further studied for biochemical test variability. Most of these patients (478 patients) had precisely 2 isolates, while 19 patients had 10 or more (up to 24) isolates of *K. pneumoniae* during the three-year study period.

### Biochemical Phenotype Descriptive Analysis

The initial *K. pneumoniae* isolates from the 2,485 patients were used to calculate the frequency of positive results for the 48 tests (Table 1). While the majority of the tests (32 out of 48) had a percentage of positive results above 95% or below 5% (and thus valuable for species identification), some were highly variable, such as TyrA (52.3% positive) and SUCT (43.5% positive).

In total, there were 900 distinct biochemical phenotypes when all 48 tests are considered, with the 10 most frequent phenotypes displayed in Table 2. The most frequent phenotype was seen in 10.8% of patients, and the top 10 phenotypes constituted 35.9% of all isolates. 551 patients (22.2%) had unique phenotypes seen in no other patient.

**Table 2:** Frequency of bionumbers of *Klebsiella pneumoniae* isolated from specimens collected January 2009-December 2011 considering the full biochemical phenotype.

Full biochemical phenotype	Discordance	Frequency	Percentage
**011011000111111110001011101110100011001000100000**	None	510	12.1
011011000111111110001**1**11101110100011001000100000	TyrA	208	5.0
0110110001111111100010111**1**1110100011001000100000	dTag	148	3.5
0110110001**0**1111110001011101110100011001000100000	GGT	129	3.1
01101100011111111000101110111010**1**011001000100000	SUCT	100	2.4
011011000111111110001**1**1110111010**1**011001000100000	TyrA, SUCT	92	2.2
0110110001111111100010111**1**111010**1**011001000100000	dTag, SUCT	89	2.1
011011000111111110001**1**11101110100011**1**01000100000	TyrA, GlyA	77	1.8
011011000111111110001**1**111**1**111010**1**011001000100000	TyrA, dTag, SUCT	75	1.8
011011000111111110001**1**1110111010**1**011**1**01000100000	TyrA, SUCT, GlyA	68	1.6
Unique phenotypes		551	13.1
Other phenotypes		2153	51.3
Total		4200	100.0

The column Discordance indicates the biochemical tests which distinguish each phenotype from the most common phenotype observed.

### Variance Analyses

#### Same-day isolates discordance analysis

361 patients were studied who had at least two isolates on the first date of *K. pneumoniae* isolation. In 248 (68.7%) patients, the two same-day isolates exhibited identical results across all 48 tests. One or two differences were seen in 79 (21.9%) of the patients, in most instances presumably representing two occurrences of the same microbial strain. Six or more discrepancies were uncommon (10 patients, 2.8%), and would likely represent in most cases co-colonization or co-infection with genetically distinct strains of *K. pneumoniae.*

Among the 79 patients with one or two discrepancies, differences were seen (in order of decreasing frequency) in: GlyA (24), TyrA (21), SUCT (17), GGT (9), CMT (6), O129R (6), lLATk (5), lMLTa (5), PryA (3), ProA (3), and dTAG (2) and single discrepancies in dMAL, CIT, LHIS, ADO, BGUR and ILATa. The remaining [31] tests were completely concordant between the two isolates.

#### Average within-patient variance index

**Figure 1:**
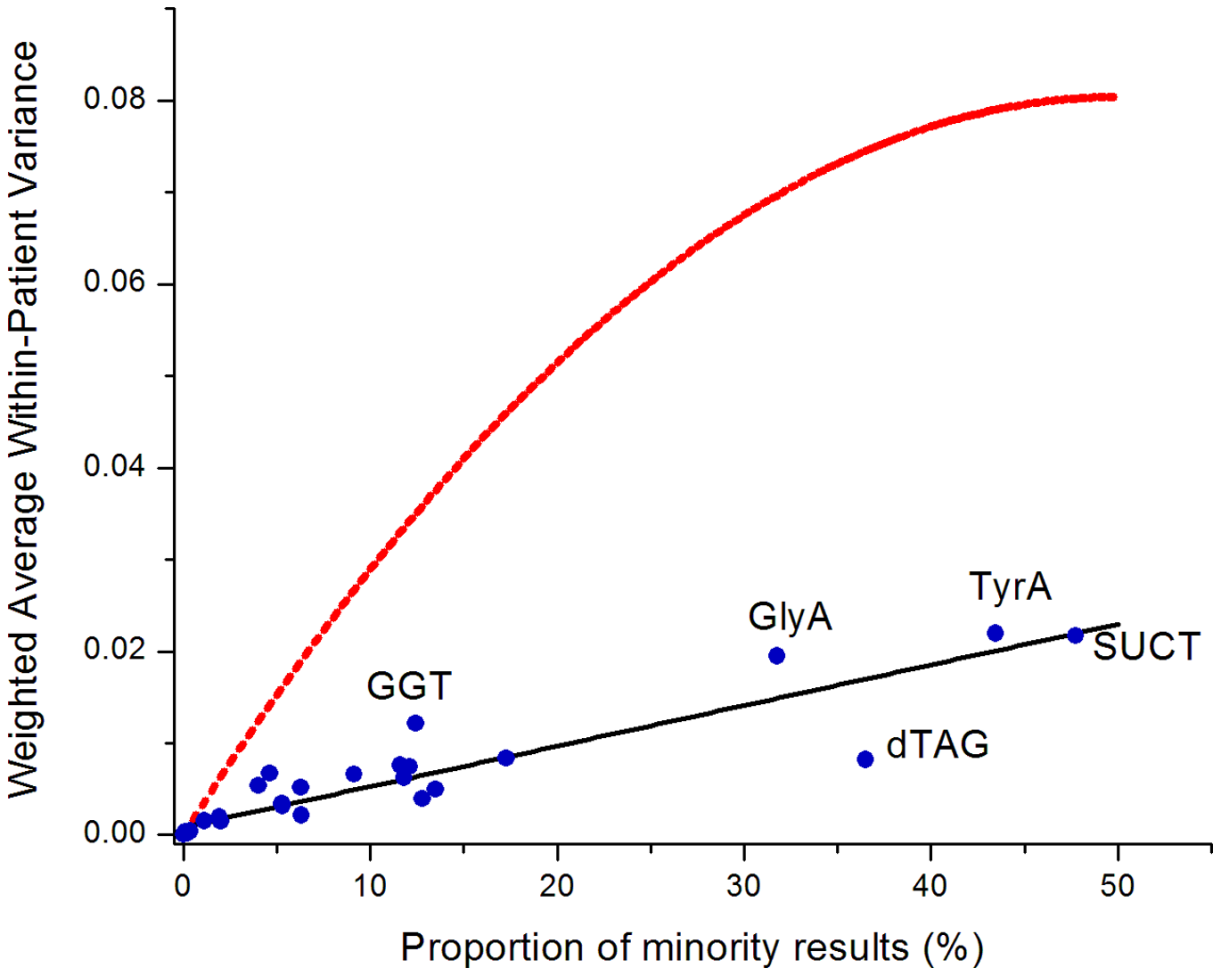
Linear regression (solid line) comparing the observed weighted average within-patient variance by proportion of minority results for 48 biochemical test results. The dashed line represents the theoretical curve that would be expected if there were no correlation between the results of a patient’s first and second isolates of *Klebsiella pneumoniae*.

Results of the average within-patient variance index are displayed in Table 1 and Figure 1. A high value indicates that there is relatively poor reproducibility of test results. Of note, the 4 biochemicals with the highest within-patient variance (and thus not very useful for reliable strain phenotyping) were precisely the same top four highlighted above in the same-day isolates discordance analysis: GlyA, TyrA, SUCT and GGT.

Conversely, a low index indicates good overall reproducibility of the test, but one cannot conclude from a low index that both positive and negative results are equally reproducible. When the proportion of minority findings is very small, the index is so heavily weighted towards the zero variance of the consistent majority results that one cannot reliably quantify how reproducible the minority result is.

Using the proportion of positive and negative results tabulated in Table 1, the dotted line in Figure 1 is a theoretical curve calculated in Microsoft Excel under the assumption that there is no relationship between the results of a patient’s first and second isolates. All of the observed values are far below the theoretical curve demonstrating that all biochemicals are much more consistent with repeat testing than one would expect by random chance, a reassuring finding with regards to the overall reproducibility of test results. For a given proportion of minority findings, biochemicals tests below the depicted regression line are relatively more reproducible than those above.

#### Between patient variance component

**Figure 2:**
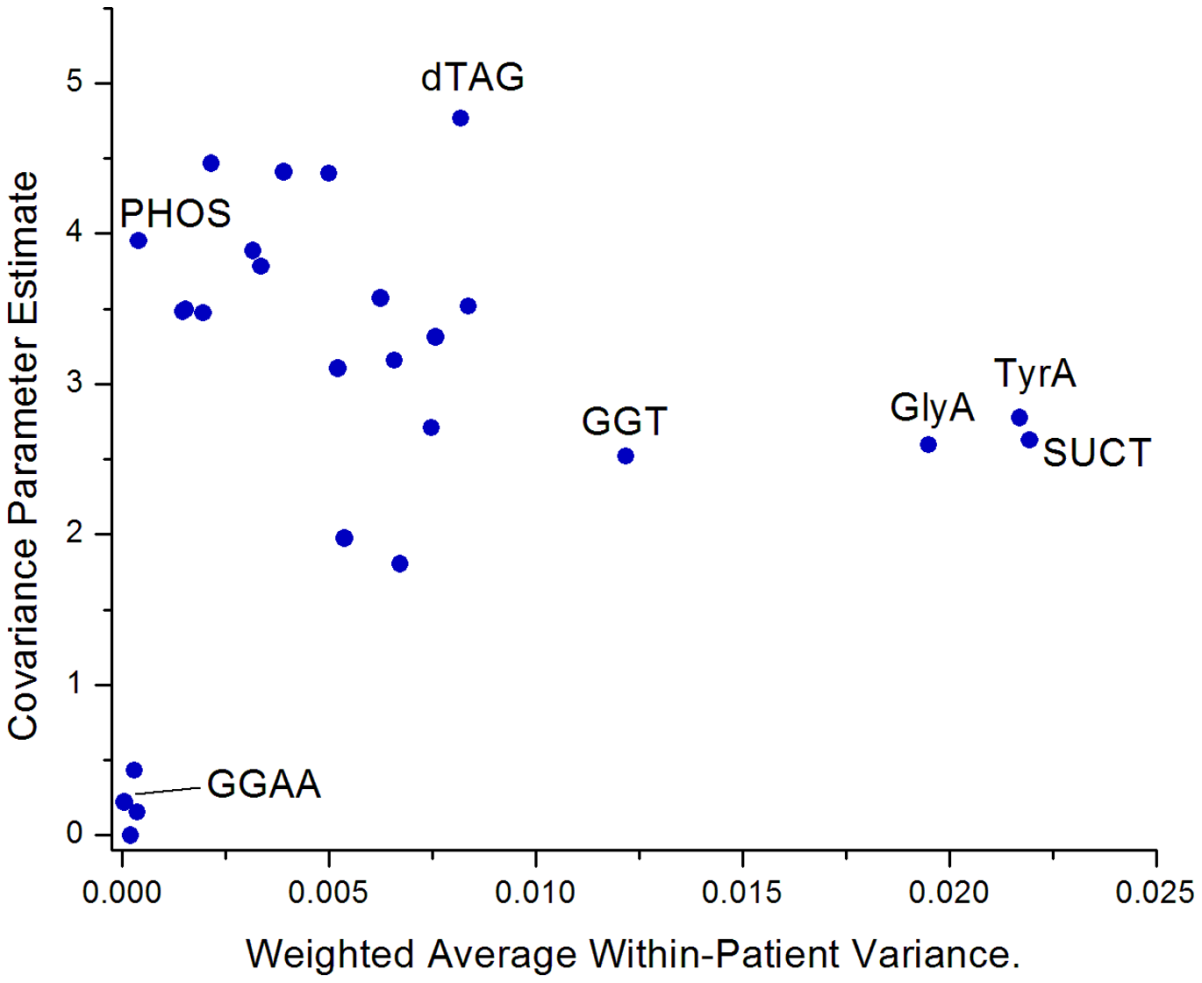
Covariance Parameter Estimate compared with observed weighted average within-patient variance.

The between patient variance component results are displayed in Table 1 and a comparison with the average within-patient variance index is shown in Figure 2. A high Covariant Parameter Estimate (CPE) suggests a high variance between patients, indicating good ability to differentiate between patients.

Of note, the four biochemicals highlighted earlier (GlyA, TyrA, SUCT, and GGT) do not stand out as remarkable in this analysis, suggesting that the observed reproducibility of these test results is consistent with their overall proportion of positive and negative results in the population. dTAG (appearing below the line in Figure 1) is noteworthy in that it displays the highest CPE (4.77) among all biochemicals.

PHOS is noteworthy in that despite the rarity of negative results (0.4%), the CPE was one of the highest observed (3.95). PHOS-negative strains thus are rare but seem to be distinctive and reproducible, a scenario similar to sorbitol example described in the Introduction. Similarly, minority results from CIT, PLE, and dMAL were rare (<2%), yet had CPEs over 3.0. In contrast, GGAA, BAlap, dMNE, and AGAL had low CPEs (<0.4) suggesting that these tests have limited ability to differentiate between *K. pneumoniae* patients.

The GLIMMIX procedure did not converge for several biochemicals because of insufficient data. With a larger data set, one would anticipate that convergence would be feasible for most biochemicals with the exception of those which are uniformly positive or uniformly negative.

### Reduced Biochemical Phenotype

The first two variance analyses presented suggest that four biochemical tests are particularly problematic, impacting a large number of patient isolates – TyrA, SUCT, GlyA, and GGT. For the first three of these, the proportion of minority results was high (47.7%, 43.5%, and 31.8% respectively) and the within-patient variance was also high (Figure 1). For GGT, the proportion of minority results (12.4%) was similar to that of several other biochemicals, but the within-patient variance was 45% higher than the next highest variance.

It would thus be useful to explore phenotype distributions in our study population results of these four biochemicals excluded from consideration. These results are depicted in Table 3, and demonstrate significant collapsing of biochemical phenotypes into fewer categories. The number of distinct phenotypes is now 522 (previously 900), while the proportion of isolates with the most common bionumber increases from 10.8% to 30.8% and the proportion of isolates with one of the ten top phenotypes increases from 35.9% to 61.2%. The number of patients with unique phenotypes decreases from 551 (13.1% of patients) to 283 (6.7%).

**Table 3:** Frequency of bionumbers of *Klebsiella pneumoniae* isolated from specimens collected January 2009–December 2011, considering the biochemical phenotype without 4 biochemical tests (GGT, TyrA, SUCT and GlyA).

Reduced biochemical phenotype	Discordance	Frequency	Percentage
**0110110001_1111110001_1110111010_011_01000100000**	None	1293	30.8
0110110001_1111110001_111**1**111010_011_01000100000	dTag	544	12.9
0110110001_1111110001_1110111010_011_01000**0**00000	0129R	171	4.1
0110110001_1111110001_**0**110111010_011_01000100000	Ure	121	2.9
0110110001_1111110001_111011**0**010_011_01000100000	MNT	108	2.6
0110110001_1111110001_111**1**111010_011_01000**0**00000	dTag, 0129R	89	2.1
0110110001_1111110001_111**1**11**0**010_011_01000100000	dTag, MNT	78	1.9
0110110001_1111110001_1110111010_011_0**0**000100000	LDC	71	1.7
0110110001_1111110001_111**1**111010_011_010**1**0100000	dTag, CMT	48	1.1
0110110001_1111110**1**01_**0**110111010_011_01000100000	ProA, Ure	47	1.1
Unique phenotypes		283	6.7
Other phenotypes		1347	32.1
Total		4200	100.0

The column Discordance indicates the biochemical tests which distinguish each phenotype from the most common phenotype observed.

Table 4 illustrates the impact of selective biochemical suppression using results from two patients, each of whom had 10 isolates of *K. pneumoniae.* If all biochemical tests are considered equivalently, Patient 1 would appear to have six distinct phenotypes with a total of 7 discordant results (out of 480 tests), and Patient 2 three phenotypes with 17 discordant results. However, if one discounts the discrepancies seen in the four gray columns, then Patient 1 has only two distinct phenotypes with a single discordant result (ProA) (out of 440 tests), while Patient 2 has three phenotypes with 11 discordant results (multiple biochemicals). The reduced biochemical phenotype thus suggests that Patient 1 in fact has multiple isolates of a single strain which exhibited a single variant result (ProA), while Patient 2 appears to have two distinct strains with a single discordant result observed in the second strain (ILATa).

**Table 4:** Heterogeneity analysis in patients with large numbers of repeat isolates of *Klebsiella pneumoniae*.

	Days	APPA	ADO	PyrA	IARL	dCEL	BGAL	H2S	BNAG	AGLTp	dGLU	GGT	OFF	BGLU	dMAL	dMAN	dMNE	BXYL	BAlap	ProA	LIP	PLE	TyrA	URE	dSOR	SAC	dTAG	dTRE	CIT	MNT	5KG	ILATk	AGLU	SUCT	PHOS	AGAL	NAGA	GlyA	ODC	LDC	LHISa	CMT	BGUR	O129R	GGAA	IMLTa	ELLM	ILATa	N_CON
Patient 1	0	0	1	1	0	1	1	0	0	0	1	1	1	1	1	1	1	1	0	1	0	1	1	0	1	1	0	1	1	1	0	1	0	0	0	1	1	0	0	1	0	0	0	1	0	0	0	0	0
	2	0	1	1	0	1	1	0	0	0	1	1	1	1	1	1	1	1	0	1	0	1	**0**	0	1	1	0	1	1	1	0	1	0	0	0	1	1	0	0	1	0	0	0	1	0	0	0	0	0
	9	0	1	1	0	1	1	0	0	0	1	1	1	1	1	1	1	1	0	1	0	1	1	0	1	1	0	1	1	1	0	1	0	**1**	0	1	1	0	0	1	0	0	0	1	0	0	0	0	0
	23	0	1	1	0	1	1	0	0	0	1	1	1	1	1	1	1	1	0	1	0	1	1	0	1	1	0	1	1	1	0	1	0	0	0	1	1	0	0	1	0	0	0	1	0	0	0	0	0
	23	0	1	1	0	1	1	0	0	0	1	1	1	1	1	1	1	1	0	1	0	1	1	0	1	1	0	1	1	1	0	1	0	0	0	1	1	0	0	1	0	0	0	1	0	0	0	0	0
	23	0	1	1	0	1	1	0	0	0	1	1	1	1	1	1	1	1	0	1	0	1	**0**	0	1	1	0	1	1	1	0	1	0	**1**	0	1	1	0	0	1	0	0	0	1	0	0	0	0	0
	29	0	1	1	0	1	1	0	0	0	1	1	1	1	1	1	1	1	0	1	0	1	1	0	1	1	0	1	1	1	0	1	0	0	0	1	1	0	0	1	0	0	0	1	0	0	0	0	0
	30	0	1	1	0	1	1	0	0	0	1	1	1	1	1	1	1	1	0	1	0	1	1	0	1	1	0	1	1	1	0	1	0	0	0	1	1	0	0	1	0	0	0	1	0	0	0	0	0
	36	0	1	1	0	1	1	0	0	0	1	1	1	1	1	1	1	1	0	1	0	1	**0**	0	1	1	0	1	1	1	0	1	0	0	0	1	1	0	0	1	0	0	0	1	0	0	0	0	0
	37	0	1	1	0	1	1	0	0	0	1	1	1	1	1	1	1	1	0	**0**	0	1	**0**	0	1	1	0	1	1	1	0	1	0	0	0	1	1	0	0	1	0	0	0	1	0	0	0	0	0
Patient 2	0	0	1	1	0	1	1	0	0	0	1	1	1	1	1	1	1	1	0	0	0	1	0	1	1	1	0	1	1	1	0	1	0	0	0	1	1	0	0	1	0	0	0	1	0	0	0	0	0
	0	0	1	1	0	1	1	0	0	0	1	1	1	1	1	1	1	1	0	0	0	1	0	1	1	1	0	1	1	1	0	1	0	0	0	1	1	0	0	1	0	0	0	1	0	0	0	0	0
	0	0	1	1	0	1	1	0	0	0	1	1	1	1	1	1	1	1	0	0	0	1	0	1	1	1	0	1	1	1	0	1	0	0	0	1	1	0	0	1	0	0	0	1	0	0	0	0	0
	0	0	1	1	0	1	1	0	0	0	1	1	1	1	1	1	1	1	0	0	0	1	0	1	1	1	0	1	1	1	0	1	0	0	0	1	1	0	0	1	0	0	0	1	0	0	0	0	0
	1	0	1	1	0	1	1	0	0	0	1	1	1	1	1	1	1	1	0	0	0	1	0	1	1	1	0	1	1	1	0	1	0	0	0	1	1	0	0	1	0	0	0	1	0	0	0	0	0
	1	0	1	1	0	1	1	0	0	0	1	1	1	1	1	1	1	1	0	0	0	1	0	1	1	1	0	1	1	1	0	1	0	0	0	1	1	0	0	1	0	0	0	1	0	0	0	0	0
	1	0	1	1	0	1	1	0	0	0	1	1	1	1	1	1	1	1	0	0	0	1	0	1	1	1	0	1	1	1	0	1	0	0	0	1	1	0	0	1	0	0	0	1	0	0	0	0	0
	1	0	1	1	0	1	1	0	0	0	1	1	1	1	1	1	1	1	0	0	0	1	0	1	1	1	0	1	1	1	0	1	0	0	0	1	1	0	0	1	0	0	0	1	0	0	0	0	0
	120	0	**0**	1	0	1	1	0	0	0	1	1	1	1	1	1	1	1	0	**1**	0	1	**1**	1	1	1	**1**	1	1	1	**1**	1	0	**1**	0	1	1	**1**	0	1	0	0	0	1	0	**1**	0	**1**	0
	120	0	**0**	1	0	1	1	0	0	0	1	1	1	1	1	1	1	1	0	**1**	0	1	**1**	0	1	1	**1**	1	1	1	**1**	1	0	**1**	0	1	1	**1**	0	1	0	0	0	1	0	**1**	0	0	0

Columns in gray represent the four biochemicals removed to create the reduced biochemical phenotype. Removal of these test results facilitates conclusions about the relatedness of patient isolates.

### Carbapenem-resistant Enterobacteriaceae

CRE are a rapidly growing threat worldwide with significant morbidity and mortality with few or no treatment alternatives [20]–[25], and the emergence and spread of these strains is a public health priority. During the study period, 24 isolates of imipenem-resistant *K. pneumoniae* were isolated from 13 patients. Four of these patients had multiple isolates (15 in total), while the remaining 9 patients had a single isolate.

With the reduced biochemical set, 10 strain phenotypes could be distinguished, shown in Table 5. Resistance phenotypes within each reduced biochemical phenotype were identical, suggestive that indeed these were multiple isolates of a single clone.

**Table 5:** Biochemical and resistance phenotypes of imipenem-resistant *K. pneumoniae.*

Strain	Biochemical discrepancies from the most common phenotype	Number of patients with CRE	Number of isolates with CRE	Number of patients with same reduced phenotype	Profile
1	PyrA, H2S, TyrA, ILATk, SUCT, ODC, CMT, O129R, ELLM	1	2	1	AMP IPM
2	TyrA, dTag, SUCT	1	1	403	AMP CAZ ETP IPM SXT
3	dTag, SUCT, CMT	1	1	25	AMP CAZ CRO IPM CIP
4	PLE, TyrA	1	1	8	AMP CAZ CRO ETP IPM GEN CIP
5	GGT, dMAL	2	2	14	AMP CAZ CRO ETP IPM GEN CIP SXT
6	GGT	1	1	827	AMP CAZ CRO ETP IPM AMK CIP SXT
7	GGT, dMal, URE, SUCT, GlyA	1	4	1	AMP CAZ CRO ETP IPM GEN CIP SXT
8	BXYL, SAC, LDC	2	3	30	AMP CAZ CRO ETP IPM GEN AMK CIP
9	ProA, TyrA, URE	2	8	80	AMP CAZ CRO ETP IPM GEN AMK CIP SXT
10	GGT, SAC, ILATk	1	1	3	AMP CAZ CRO ETP IPM GEN AMK CIP SXT

Number of patients  = 13, Number of isolates  = 24. The antibiotic code indicates that the organism is non-susceptible to the indicated agent. AMP = Ampicillin, CAZ = Ceftazidime, CRO = Ceftriaxone, ETP = Ertapanem, IPM = Imipenem, GEN = Gentamicin, AMK =  Amikacin, CIP = Ciprofloxacin, SXT = Trimethoprim/Sulfamethoxazole.

Of note, Strain 6 is amikacin-resistant yet gentamicin-susceptible, a rare resistance phenotype suggestive of the enzyme aac6'1b [26], infrequently seen in the United States, but seen recently in increasing numbers in this facility.

### Cluster Detection

A comparison of the WHONET-SaTScan cluster findings when utilizing full (“F”, 2 clusters detected) and reduced (“R”, 3 clusters detected) biochemical phenotypes is shown in Table 6. Resistance phenotypes within each of these clusters were concordant. One event was detected by both methods - Clusters F1 (4 patients) and R2 (8 patients, including all 4 patients in Cluster F1). In this event, the reduced biochemical phenotype identified four additional patients missed with the full biochemical profile. The clinical and epidemiological significance of Clusters F2, R1, and R3 (as well as Clusters F1 and R2) cannot be ascertained with certainty without additional epidemiological and perhaps molecular investigation, but the statistical findings themselves if found in real-time could have prompted such investigation.

**Table 6:** Comparison of the WHONET-SaTScan cluster findings when utilizing full (F) and reduced (R) biochemical phenotypes of *Klebsiella pneumoniae*.

Cluster	Cluster biochemical phenotype	Start date	First signal	End date	RI - Highest	Number of patients
F1	001011000111111110101111111111101011101100101010	8/23/2010	8/26/2010	8/26/2010	3842	4
F2	011011000111111110001111111110100011001000100000	5/5/2011	5/13/2011	5/13/2011	1168	6
R1	0010110001_1111110101_1110111110_011_01000100000	7/14/2010	7/25/2010	7/25/2010	385	4
R2	0010110001_1111110101_1111111110_011_01100101010	8/13/2010	8/26/2010	9/17/2010	5165	8
R3	0110110001_1111110001_1110111010_011_11000100000	7/9/2011	7/9/2011	7/9/2011	2342	2

RI = Recurrence Interval in days.

## Discussion

Early detection of emergent threats is critical to effective containment efforts, but the data management and analytical tools available to microbiologists, infection control staff, and public health authorities are limited [27], [28]. In this study, we explored how the use of routinely available biochemical test result details, typically ignored by microbiologists, can enrich the delineation of microbial strains within a species and how this information can be used to monitor evolving populations and improve detection of outbreaks [29], [30].

The heterogeneity of biochemical phenotypes is evident in product inserts [9] and in our observed results (Table 1). However, such presentations do not distinguish between stable characteristics which vary between distinct strains and “micro-variability” seen with repeat testing of a specific strain due to issues of test performance or in the biological expression of phenotypes. Consequently, we explored algorithms that could be applied to any organism and to many types of microbiological tests to facilitate the application of routine microbiological data to the study of evolving microbial populations. The first two variance analysis approaches are conceptually similar, focusing on an empiric description of within-patient discrepant findings. The most frequent reason for test discordance in same-day isolates is limitations of the test method, though in some instances, discordance could be due to mutations or co-infection/colonization with distinct strains of the same bacterial species.

An advantage of the same-day isolates approach is that the calculations are simple to perform and communicate. A limitation of the same-day approach is the relatively limited number of patients, isolates, and rare test findings included in the analysis.

The average within-patient analysis more effectively utilizes the complete database to quantify variance estimates. As depicted in Figure 1, this approach permits a general appreciation as to how much the observed variability can be attributed to the relative proportion of minority findings in the population (one would expect biochemicals to the right of the figure to have higher variances because of the frequency of both positive and negative results) versus how much can be attributed to intrinsic biological and test performance variability (at a given proportion of minority findings, biochemicals above the regression line are more variable than biochemicals below the line).

These two variances highlighted four biochemicals as “nuisance” tests impacting a large number of patients. While these tests are of limited value in the identification of *K. pneumoniae*, the Vitek Product Information manual [9] confirms that their results are valuable for other Gram-negative rods justifying the vendor's inclusion of these biochemicals in the Gram-negative test panel. For example, the Vitek product manual indicates that TyrA is generally consistent (95–100%) for *S. sonnei* and *A. baumannii*, while SUCT is consistent for *E. meningoseptica* and *A. baumannii*. In this context it is important to note that our evaluation of the different biochemical tests are valid for epidemiological distinction between different patients that all have *K. pneumoniae;* and for a different pathogen, we would expect that a completely different set of the 48 biochemical tests would be useful. Hence the variance component analyses must be repeated for each pathogen of interest.

The between patient variance component estimates provide a different type of insight into the results than offered by the previous two approaches. For a biochemical test to be a useful epidemiological marker, it is not enough to have a small within patient variance, a large between patient variance component is also needed. For rare minority results (*e.g.* sorbitol-negative results in *E. coli*) to serve as reliable strain markers, stability is critical, yet cannot be assessed by the previous variance analyses. Our between patient variance component findings would suggest that PHOS, CIT, PLE, and dMAL could potentially prove useful as strain markers for *K. pneumoniae*, whereas minority findings in GGAA, BAlap, dMNE, and AGAL are too similar across patients to be of great use as epidemiological markers. In short, the first two variance analysis approaches described seem to be particularly well-suited for identifying “nuisance” biochemicals which exhibit significant within-patient test variability, while between patient variance component is particularly valuable for characterizing the utility of biochemical tests with rare phenotypes. dTAG was noteworthy in that results were relatively consistent within a strain despite the high proportion of both positive and negative results.

By excluding the four most problematic biochemicals from consideration, results suggest that strain recognition from phenotype results becomes more robust (CRE *K. pneumoniae* example) and can improve the timeliness and specificity of cluster detection (WHONET-SaTScan example). In both examples, the relevance of the phenotypic designations was supported by the consistency of resistance phenotypes among strains. In the CRE example, it is noteworthy that Strains 1 and 7 had reduced biochemical phenotypes previously unrecognized at this healthcare facility. To explore whether the carbapenem resistance gene in these two strains evolved were imported into the facility from an external origin, it would be relevant to compare the biochemical results of these two isolates (as well as the others) to the biochemical phenotypes of CRE identified elsewhere.

Biochemical phenotypes often reflect stable, ancient chromosomal strain characteristics, while antimicrobial resistance phenotypes frequently reflect relatively recent mutations and gene acquisitions. The use of both types of these microbiological test results together could thus perhaps offer something close to a phenotypic “fingerprinting” for strain tracking which could be confirmed further with more time-consuming and expensive molecular typing techniques. Microbial phenotypes are generated daily worldwide to support routine clinical care, but are generally ignored by clinicians and epidemiologists. Through application of the approaches described in this work, it should be possible to improve the timely identification of and response to evolving microbial threats.

## References

[1] Stelling J, Yih WK, Galas M, Kulldorff M, Pichel M, et al (2010) Automated use of WHONET and SaTScan to detect outbreaks of *Shigella spp.* using antimicrobial resistance phenotypes. Epidemiol Infect 138: 873–883. 19796449 10.1017/S0950268809990884 PMC4093803

[2] Gaynes R, Edwards JR (2005) Overview of nosocomial infections caused by gram-negative bacilli. Clin Infect Dis 41: 848–854. 16107985 10.1086/432803

[3] Wright MO, Perencevich EN, Novak C, Hebden JN, Standiford HC, et al (2004) Preliminary assessment of an automated surveillance system for infection control. Infect Control Hosp Epidemiol 25: 325–332. 15108731 10.1086/502400

[4] O'Brien TF, Stelling J (2011) Integrated Multilevel Surveillance of the World's Infecting Microbes and Their Resistance to Antimicrobial Agents. Clin Microbiol Rev 24: 281–295. 21482726 10.1128/CMR.00021-10 PMC3122493

[5] Burke JP (2003) Surveillance, reporting, automation, and interventional epidemiology. Infect Control Hosp Epidemiol 24: 10–12. 12558229 10.1086/502108

[6] Huang SS, Yokoe DS, Stelling J, Placzek H, Kulldorff M, et al (2010) Automated detection of infectious disease outbreaks in hospitals: a retrospective cohort study. PLoS Med 7: e1000238. 20186274 10.1371/journal.pmed.1000238 PMC2826381

[7] Jackson ML, Baer A, Painter I, Duchin J (2007) A simulation study comparing aberration detection algorithms for syndromic surveillance. BMC Med Inform Decis Mak 7: 6. 17331250 10.1186/1472-6947-7-6 PMC1821319

[8] Hansen DS, Aucken HM, Abiola T, Podschun R (2004) Recommended test panel for differentiation of *Klebsiella species* on the basis of a trilateral interlaboratory evaluation of 18 biochemical tests. J Clin Microbiol 42: 3665–3669. 15297514 10.1128/JCM.42.8.3665-3669.2004 PMC497635

[9] bioMérieux (2010) Vitek 2 System. Product Information.

[10] March SB, Ratnam S (1986) Sorbitol-MacConkey medium for detection of *Escherichia coli* O157:H7 associated with hemorrhagic colitis. J Clin Microbiol 23: 869–872. 3519658 10.1128/jcm.23.5.869-872.1986 PMC268739

[11] Werber D, Bielaszewska M, Frank C, Stark K, Karch H (2011) Watch out for the even eviler cousin-sorbitol-fermenting *E coli* O157. Lancet 377: 298–299. 21256378 10.1016/S0140-6736(11)60090-1

[12] O'Brien TF, Stelling JM (1995) WHONET: an information system for monitoring antimicrobial resistance. Emerg Infect Dis 1: 66. 10.3201/eid0102.950209 PMC2626837 8903165

[13] O'Brien TF, Stelling JM (1996) WHONET: removing obstacles to the full use of information about antimicrobial resistance. Diagn Microbiol Infect Dis 25: 162–168. 8937840

[14] Stelling JM, O'Brien TF (1997) Surveillance of antimicrobial resistance: the WHONET program. Clin Infect Dis 24 Suppl 1S157–168. 8994799 10.1093/clinids/24.supplement_1.s157

[15] Sharma A, Grover PS (2004) Application of WHONET for the surveillance of antimicrobial resistance. Indian J Med Microbiol 22: 115–118. 17642708

[16] Witte JS, Greenland S, Kim LL, Arab L (2000) Multilevel modeling in epidemiology with GLIMMIX. Epidemiology 11: 684–688. 11055630 10.1097/00001648-200011000-00012

[17] Corporation S, Kulldorff M (2011) The Glimmix Procedure. http://www.ats.ucla.edu/stat/sas/glimmix.pdf, Accessed 8 October 2013.

[18] SaTScan, version 9.1.1. Available: http://www.satscan.org, Accessed 2013 Oct 8.

[19] Viñas MR, Tuduri E, Galar A (2013) Laboratory-based prospective surveillance for community outbreaks of *Shigella spp.* in Argentina using a space-time permutation test. PLOS Negl Trop Dis. In press. 10.1371/journal.pntd.0002521 PMC3861122 24349586

[20] Magiorakos AP, Suetens C, Monnet DL, Gagliotti C, Heuer OE (2013) The rise of carbapenem resistance in Europe: just the tip of the iceberg? Antimicrob Resist Infect Control 2: 6. 23410479 10.1186/2047-2994-2-6 PMC3691711

[21] Johnson AP, Woodford N (2013) Global spread of antibiotic resistance: the example of New Delhi metallo-beta-lactamase (NDM)-mediated carbapenem resistance. J Med Microbiol. 10.1099/jmm.0.052555-0 23329317

[22] Hussein K, Raz-Pasteur A, Finkelstein R, Neuberger A, Shachor-Meyouhas Y, et al. (2013) Impact of carbapenem resistance on the outcome of patients' hospital-acquired bacteraemia caused by *Klebsiella pneumoniae*. J Hosp Infect. 10.1016/j.jhin.2012.10.012 23313086

[23] Tanne JH (2012) Resistance of enterobacteria to carbapenem antibiotics is a global crisis. BMJ 344: e1646. 22396112 10.1136/bmj.e1646

[24] Nordmann P, Dortet L, Poirel L (2012) Carbapenem resistance in *Enterobacteriaceae*: here is the storm! Trends Mol Med. 18: 263–272. 10.1016/j.molmed.2012.03.003 22480775

[25] Braykov NP, Eber MR, Klein EY, Morgan DJ, Laxminarayan R (2013) Trends in Resistance to Carbapenems and Third-Generation Cephalosporins among Clinical Isolates of *Klebsiella pneumoniae* in the United States, 1999–2010. Infect Control Hosp Epidemiol 34: 259–268. 23388360 10.1086/669523

[26] Tolmasky ME, Chamorro RM, Crosa JH, Marini PM (1988) Transposon-mediated amikacin resistance in *Klebsiella pneumoniae* . Antimicrob Agents Chemother 32: 1416–1420. 2848445 10.1128/aac.32.9.1416 PMC175879

[27] Brisse S, Fevre C, Passet V, Issenhuth-Jeanjean S, Tournebize R, et al (2009) Virulent clones of *Klebsiella pneumoniae*: identification and evolutionary scenario based on genomic and phenotypic characterization. PLoS One 4: e4982. 19319196 10.1371/journal.pone.0004982 PMC2656620

[28] Levert M, Zamfir O, Clermont O, Bouvet O, Lespinats S, et al (2010) Molecular and evolutionary bases of within-patient genotypic and phenotypic diversity in *Escherichia coli* extraintestinal infections. PLoS Pathog 6: e1001125. 20941353 10.1371/journal.ppat.1001125 PMC2947995

[29] Alves MS, Dias RC, de Castro AC, Riley LW, Moreira BM (2006) Identification of clinical isolates of indole-positive and indole-negative *Klebsiella spp* . J Clin Microbiol 44: 3640–3646. 16928968 10.1128/JCM.00940-06 PMC1594763

[30] O'Brien TF, Ross DG, Guzman MA, Medeiros AA, Hedges RW, et al (1980) Dissemination of an antibiotic resistance plasmid in hospital patient flora. Antimicrob Agents Chemother 17: 537–543. 7396449 10.1128/aac.17.4.537 PMC283828

